# Continuation of a Memoir on the Morbific Elongation of the Tongue out of the Mouth

**Published:** 1801-12

**Authors:** Pierre Lassus, G. Bellamy


### *Continuation of a Memoir on the morbific Elongation of the Tongue out of the Mouth.* By PIERRE LASSUS.

WE read in a Treatise of Surgery, published in 1782, by Le Blanc, a single observation so much the more useful, that by considering it in its proper point of view, it will serve to prove the solidity of the principles stated in this Memoir. — The daughter of a basket-maker of Orleans, was attacked at the age of three years with convulsions, which caused the tongue to protrude from the mouth, and prevented the patient from drawing it in again. She bore this hideous inconvenience for fourteen years. In the month of April 1772, Le Blanc was desired to see her; she was then seventeen years old. The tongue was out of the mouth the length of three fingers breadth, and was more than an inch thick; it was covered with seven or eight small ulcers, and under were three or four ulcerated protuberances. The girl was disgusting to all who saw her; the incisive teeth of the lower jaw were turned outwards, and covered with tartar. The author of this observation thought, that by enclosing the tongue in a small bag of fine cloth, returning it into the mouth, and keeping it there some time with a bridle, it would diminish in size; that the muscles which serve to draw it towards the bottom of the mouth, having been too much extended for 14 years, would, in the end, regain their spring; and that the saliva, which continually softened the ulcers, would alone suffice to clean and heal them. According to this idea he enclosed the tongue in a small bag of cloth, to the angles of which he attached a bridle made with brass wire, which was to be applied under the chin, as a point of solid support. Ribbons attached to the same bridle were conducted from before, backwards, to be crossed on the nape, and then be brought forwards to be tied on the forehead. This apparatus was taken off, evening and morning, to wash the bag, and rince the patient's mouth with wine. In the space of four days the tongue diminished considerably in size, resumed its place, and the ulcers cleansed. From that time the bridle was unnecessary, and the tongue came no more out of the mouth; but as the incisive teeth of the lower jaw, which were thrown from their alveoli, were inserted in the inferior lip, and prevented its being applied to to the ſuperior, eſpecially when the girl wiſhed to ſhut her mouth, it was neceſſary to extract them; then the patient was for ever freed of any inconvenience which had rendered her incognizable.* The advice of encloſing the tongue in a kind of fine linen bag attached to a braſs wire, which draws in the manner of a bridle under the chin, has been given as a proper means to favour the cure of tranſverſe wounds of this organ.† Le Blanc thought that the ſame proceſs might be uſefully employed in the cure of the baſket-maker's daughter of Orleans. It is, in fact, to the retropulſion performed by the action of this bandage upon the extremity of the tongue, that this girl owed her cure; it does honour to the underſtanding and ſagacity of the profeſſional man who directed it. But in a child the ſame means would not be ſo efficacious, the tongue having neither the ſame ſize nor elongation; and not being able to furniſh a point of ſupport ſufficiently ſolid, the commiſſure of the lips would be an obſtacle to the action of this bandage, which in acting too feebly from before backwards, could not force back the tongue and keep it in the mouth. Beſides, if we pay attention to the indocility of an infant whom this bridle inconveniences, and which neceſſarily keeps the mouth partly open, it will be ſeen that it cannot always fulfil an object to which it was not, in the firſt place, deſtined by its author. It reſults from the moſt elementary notions of anatomy, that deglutition cannot be performed, or at leaſt but very badly, when the mouth is open; it is neceſſary to execute that natural action, that the jaws ſhould be brought together; then the os hyoides, which is a ſupport to the tongue, riſes with the ſuperior part of the larynx, whilſt the baſe of the tongue carried backwards, acts againſt the portion of aliment which is directed towards the pharynx; it is thus that the elevating muſcles of the jaw eſſentially contribute to deglutition. It is then true, that to cure in new-born infants the diſeaſe in queſtion, the child muſt not only be prevented from ſucking, but the jaws muſt alſo be kept together, to direct as much as poſſible the movements of the tongue during deglutition; in a ſenſe contrary to that which it has hitherto maintained, and which it would continue to preſerve by ſucking the nurſe's breaſt. I ſhall finiſh this Memoir by the recital of a fact, the more important to be known, as it will prove with what facility this diſeaſe may be cured when it has even made the moſt conſiderable advances.

There lives in Paris, in a houſe for females, called la Salpêtriere, a woman forty years old, born with the tongue elongated,

---

* Precis d'Operations de Chirurgie, par Le Blanc, p. 17.

† Memoires de l'Academie de Chirurgie, Tom. III, p. 418.

ted from the mouth. She bore this inconvenience for thirty years, ſpeaking but with difficulty, and not even able to pronounce ſeveral ſyllables; the teeth of the lower jaw were thrown outwards by degrees, the lip reverſed; and the ſaliva, no longer retained in the mouth, fell continually upon the chin and breaſt. The impreſſion of heat or cold on the tongue was very painful, and this organ was commonly redder, and more tumefied during winter than ſummer. When this woman was thirty years old, ſhe conſulted the late Louis, Secretary of the Academy of Surgery, who declared to her at firſt, that there was no hope of cure; but a few days after he ſaw the patient again, and adviſed her, after having read an obſervation to be found in Galen, to encloſe the tongue in cloths immerſed in the juice of lettuce. In fact, we read in the works of the Phyſician of Pergamus,* that an old man who had the tongue ſwollen by a catarrhal or pituitous affection, was cured by taking a purgative, and by holding in his mouth the acrid and irritating juice of the wild lettuce, (Thridacene) a plant which Galen, as well as Theophraſtus and Dioſcorides carefully diſtinguiſh from the cultivated lettuce, which they called Thridax. The woman of the Salpêtrière followed the advice which had been given her; ſhe wetted her tongue conſtantly, and for ſix months entirely, with the juice of the cultivated lettuce. Galen would probably have recommended that of the wild, as a repercuſſive remedy by its roughneſs. Be that as it may, the tongue, which was dried up, ſoftened again, and became more humid. The continued uſe of this inſipid remedy, applied cold for a great length of time, and conſtantly renewed, inſenſibly performed the diminution of ſize in this organ. The woman is now perfectly cured, but ſtill experiences a difficulty in ſpeaking, altho' the tongue has been completely retained in the mouth for ſeven or eight years. The ſkin of the chin, which for thirty years was covered by the tongue, is alſo a little redder and thicker than in its natural ſtate.

It reſults from all the Obſervations which I have related in this Memoir, that the elongation of the tongue out of the mouth is a chronic diſeaſe, which differs eſſentially from every kind of inflammatory tumor of which this organ is ſuſceptible from very various cauſes. I have already ſaid, that it is not eſſential to the diſeaſe of which I ſpeak, that the tongue ſhould be very much tumefied; that accident is manifeſted but conſecutively;

* Methodus Medendi, Lib. XIV. cap. 8. De Differentiis Morborum, cap. 9.

cutively; and it is not becaufe this organ is primitively more voluminous, or longer than it ought to be, that it becomes difplaced, but from a caufe which is not always the fame, as we have feen in the preceding obfervations: Yet, when this accident does take place, when the tumefaction is fuch that it is an obftacle to the reduction of the difeafed part, it does no lefs prefent a curative indication, which it is ufeful not to lofe fight of. It may be conceived, that under different circumftances, according as the difeafe is recent or of long ftanding, the tongue more or lefs tumefied, or dried up by its long expofure to the air, lotions or gargarifms fimply emollient, or a little ftimulant, according to the variable ftate of the affected part may be ufefully employed. By a like procefs, Scultel* cured in a few days, a man who in confequence of the inconfiderate ufe of mercury by friction, had for four months the tongue very volumnious, and hanging out of the mouth the length of four fingers breadth. By ftimulating it with an irritating powder compofed of pepper, ginger, muftard, and falt, it foon became more moveable, and lefs hard. It could be replaced, but it immediately came out of its natural place again. By making fcarifications on its inferior furface, it detumefied fo as to be for ever after retained in the mouth. It is thus that the application of leeches, which produced at the fame time a depletion and irritation on the tumefied tongue, appeared to me to be fuitable in the cafe where I employed it: But when in a new-born child the point of the tongue, not fwollen, protrudes but a few lines out of the mouth, when the difeafe as yet fhews itfelf under the appearance of a flight affection, which draws but little attention from the parents, this vicious difpofition may be efficacioufly remedied by exciting on the organ of fpeech a flight irritation, as we remedy the elongation of the uvula by ftimulating it with powdered alum or pepper. This firft refource, which it may be ufeful to repeat, fhould not occafion the neglect of applying a fling to confine the jaws together, to oppofe the exit of the tongue, the turning of the os hyoides forwards, and the ftretching of the mufcles, which ferve to fupport thefe parts in their natural fituation. In fine, it is important not to take for Cancer the difeafe in queftion, when it is inveterate; or from a falfe view, to cut off the protuberant extremity of the organ of fpeech.

G. BELLAMY.

---

* Obf. 17, Append. Obf.

